# Potential Hybridization between Two Invasive Termite Species, *Coptotermes formosanus* and *C. gestroi* (Isoptera: Rhinotermitidae), and Its Biological and Economic Implications

**DOI:** 10.3390/insects8010014

**Published:** 2017-01-25

**Authors:** Nan-Yao Su, Thomas Chouvenc, Hou-Feng Li

**Affiliations:** 1 Department of Entomology and Nematology, Ft. Lauderdale Research and Education Center, University of Florida, Ft. Lauderdale, FL 33314, USA; tomchouv@ufl.edu; 2 Department of Entomology, National Chung Hsing University, 145 Xingda Rd., Taichung 40227, Taiwan; houfeng@nchu.edu.tw

**Keywords:** termite, introgression, hybrid vigor, social insect, structural pest

## Abstract

The Asian subterranean termite, *Coptotermes gestroi*, is a tropical species but has increasingly been collected from the subtropics in recent years, making it sympatric to the Formosan subterranean termite, *C. formosanus* in at least three areas, Taiwan, Hawaii, and Florida. Simultaneous flights by these two species were observed since 2013 in South Florida, during which interspecies tandems were observed. Laboratory mating of *C. formosanus* and *C. gestroi* alates produced hybrid incipient colonies of larger population size. Studies are underway to examine the presence in the field of hybrid colonies in sympatric areas of Taiwan and Florida. Other biological characteristics of *C. formosanus* × *C. gestroi* hybrids being studied include temperature tolerance and preference, colony growth rate, wood-consumption rate, and reproductive fertility. This current research aims to determine the potential establishment of a hybrid termite population in south Florida and Taiwan. It investigates the risk of introgressive hybridization in field populations, with an emphasis on its potential ecological, evolutionary, and economic consequences.

## 1. Introduction

Of the >3000 termites species recognized globally, 80 are known to cause serious damage [1], and the genus *Coptotermes* contains the largest number (18 spp.) among these serious pest species [2]. A recent study estimated that there are approximately 25 valid *Coptotermes* species in the world and most of them are pest in their native and introduced areas [3]. However, only two *Coptotermes* species are successful invaders: the Formosan subterranean termite *C*. *formosanus* and the Asian subterranean termite *C. gestroi* [3,4]. The expansion of the modern distribution range of these two species results from their association with human activity [5,6,7] which makes them two of the most important termite pests in the world that account for much of the $32 billion annual economic impact caused by subterranean termites [2]. *Coptotermes formosanus* is primarily found in subtropical and temperate regions while *C. gestroi* is found throughout the tropics, and it was estimated that the two allopatric species evolved separately for 15–20 million years [7]. In recent years, however, *C. gestroi* expanded its range and has increasingly been collected from the subtropics, making it now sympatric to *C. formosanus* in three areas, Taiwan, Hawaii, and Florida [8].

## 2. Sympatric Areas

### 2.1. Coptotermes in Taiwan

**Figure 1:**
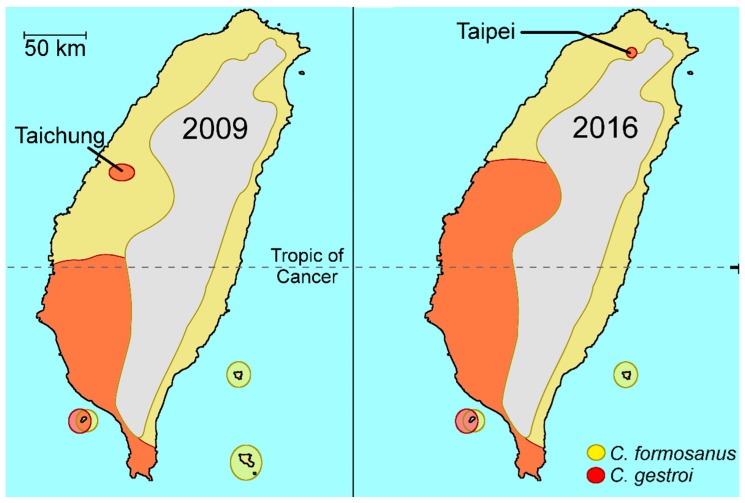
Distribution of *C. gestroi* and *C. formosanus* in Taiwan. Limited distribution of *C. gestroi* was reported in 2009 but it is frequently encountered in central and southern Taiwan. The first *C. gestroi* infestation in Taipei is reported in 2016 [12].

*Coptotermes formosanus* was the only species of this genus reported from Taiwan until 2003 when multiple specimen of *C. gestroi* were collected from central and southern parts of the island [9]. However, *C. gestroi* was probably present much earlier in Taiwan as the controversy surrounding the identification and naming of *C. formosanus* in the early 1900s probably involved specimens of both species [10]. Taiwan lies across the Tropic of Cancer, and while most of the *C. gestroi* were collected in the tropical Taiwan, *C. formosanus* have been reported from lowland area throughout the island [11]. A single collection of *C. gestroi* was reported from central Taiwan by Tsai and Chen [9], but a recent survey of the island showed *C. gestroi* has been frequently found in both central and northern Taiwan and one housed infestation was reported in 2016 in Taipei (Figure 1) [12]. The northern expansion of the *C. gestroi* distribution in Taiwan could be a recent event, and it currently appears that the entire western lowland of the island is a sympatric area for these two species. Li et al. [11] also suggested the overlapping of flight seasons (if not the same date) by alates of both species at the same location in Chiayi, but interspecies mating of these two species was not investigated. Independently, a recent synonymization of *Coptotermes* species from Hainan Island also suggests that both *C. gestroi* and *C. formosanus* are present in this area [13].

### 2.2. Coptotermes in Hawaii

*Coptotermes formosanus* was first reported in the early 1900s in Hawaii [14], and was probably introduced through the sandalwood trade from China in the 19th century [15]. It is currently found in all major islands of Hawaii. Plant quarantine records at Port of Honolulu showed that *C. vastator* (=*C. gestroi*) was found from banana stumps in shipment from Philippines as early as 1918 [16], but it did not establish there as readily as *C. formosanus*, most likely due to the subtropical climate. Weesner [17] reported specimen collected in 1963 from Kaimuki, Honolulu as *C. vastator* (=*C. gestroi*), but *C. gestroi* was not reported again until 1999 when a few infestations were found in and near Ewa Beach of Oahu [18] and since then the infestation has slowly expanded from there to nearby communities [19]. Due to the distance (several miles) between Kaimuki and Ewa Beach, the lack of any discovered infestations between these two locations, and the absence of any discoveries after 1963 until 1999, it is believed that the recent *C. gestroi* infestations result from different and more recent introduction(s) [20]. The *C. gestroi* infestation found in Ewa Beach in 1999 may therefore represent the first establishment of this species in Hawaii.

### 2.3. Coptotermes in Florida

**Figure 2:**
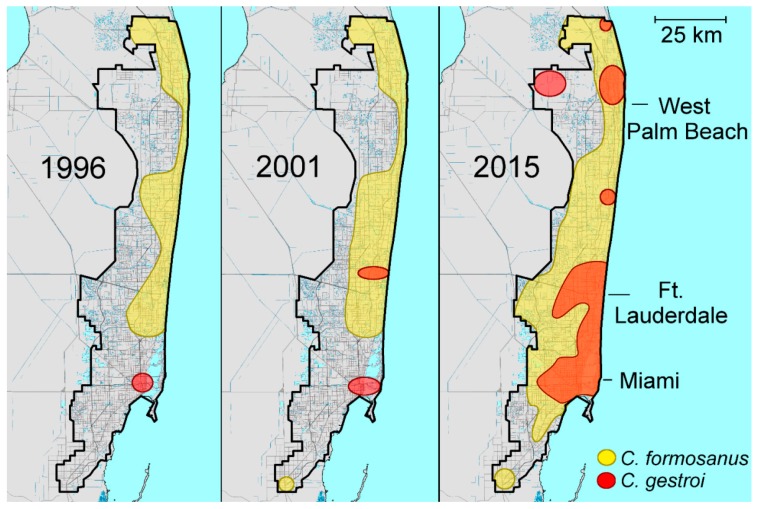
Establishment of *C. gestroi* and *C. formosanus* in Metropolitan southeastern Florida over time [24].

Since its first report in Southeastern Florida in 1980, *C. formosanus* has been found in all major urban areas of Florida [21] with North Miami Beach as the southern boundary until 2001 when a few isolated populations were reported from Homestead (Figure 1). *Coptotermes gestroi* was first reported in 1996 near the port of Miami where *C. formosanus* was absent [22], hence, they remained allopatric in Florida until 2001 when a single alate of *C. gestroi* was collected in Broward County (Figure 2) [23]. Since then, land-based *C. gestroi* infestations have been found in more locations from the lower Florida Keys to Palm Beach County. Currently, *C. gestroi* is sympatric with *C. formosanus* in Dade, Broward and Palm Beach County, a tri-county metropolitan area inhabited by ≈30% of the Florida population [24].

Even in the tri-county area where these two species are sympatric, there had been a distinct difference in their flight seasons and no simultaneous flight was observed until 2012. Alate collection records up until then showed that *C. gestroi* alates swarmed from February to mid-April and *C. formosanus* alates from mid-April to June. In fact, according to Scheffrahn [25], “…as soon as *C. formosanus* begins its first flights, those of *C. gestroi* cease…” In 2013, however, Chouvenc et al. [8] observed two events of simultaneous flights by these two species in Broward County. A more thorough survey carried out in 2014 at the same location revealed that *C. gestroi* alates swarmed from late February to early June, while *C. formosanus* swarmed from mid-March to mid-June. Alates of these two species swarmed on the same nights 24 times between 18 March and 9 June, with five events of major simultaneous swarming, i.e., >100 alates of both species collected from a light trap [8].

## 3. Hybridization

### 3.1. Observations in Coptotermes

Interspecies tandems between *C. formosanus* and *C. gestroi* alates were observed in the field during the simultaneous swarming events of 2013 and 2014 in Florida [8]. In 2015 and 2016, multiple major simultaneous swarming events were also observed and interspecies mating occurred in the field every year since 2013 [26]. Interspecies mating pairs were collected from the field during these events and reared in the laboratory. Mating of these two species produced hybrid incipient colonies with more individuals than parent colonies after the first year [8]. COII barcoding and microsatellite genotyping confirmed the hybridization of heterospecific incipient colonies. While interspecies matings were observed in the field and these matings produced successful colonies in the laboratory, it is unknown if hybrid populations are established in the field and if gene introgression has taken place in potential hybrid zones in Taiwan, Hawaii and Florida. We are currently investigating the population genetics of *Coptotermes* samples collected from sympatric zones of Taiwan and Florida to determine if hybrid populations are present in the field. Discovery of hybrid populations, however, does not necessarily imply introgressive hybridization among populations of these two species, because the fertility of F_1_ alates produced by hybrid colonies is unknown.

### 3.2. Hybridization in Social Hymenoptera

Hybridization events in social insects were previously observed, especially in social hymenopterans. However reduced fertility or sterility is often one of the fitness cost imposed on hybrid populations. Hybridization is common among many ant species [27], but many did not produce F_1_ queens (*Lasius jensi* × *L. umbratus*) [28] or only produced infertile individuals (*L. alienus* × *L. niger*, *L. latipes* × *L. claviger, Temnothorax unifasciatus* × *T. nigericeps*) [29,30,31] or reduced fertility in F_1_ queens (*Pogonomyrmex barbatus* × *P. rugosus*, *Pogonomyrmex barbatus* × *P. rugosus*, *T. parvulus* × *T. lichtensteini*) [32,33]. Social hybridogenesis is known to produce non-hybrid reproductive caste in the hybrid zone of two fire ant species, *S. geminata* and *S. xyloni*, with only worker castes being hybrid [34]. Despite the restriction imposed on hybrid F_1_ queens, however, some hybrid ants gain advantage over non-hybrid populations. In the case of *S. geminata* × *S. xyloni*, the ability to produce hybrid workers with non-hybrid reproductive may, in the absence of conspecific mates, provide an option for queens to form a viable colony with hybrid workers that survived better in competitive encounters than the parent species [34,35]. Hybrid imported fire ants, *S. invicta* × *S. richteri*, are known to outcompete native species in disturbed habitats [36], and are more tolerant to low temperatures than either parent [37]. Potentially, hybrid ants are capable of occupying marginal habitats not utilized by parent species [38].

Being haplodiploids, cross-mated ant queens produce pure-species males, and hybrid male ants are rarely found [27]. Due the non- or reduced fertility associated with hybrid F_1_ queens and mostly pure-species males, genetic introgression has not been observed in hybrid zones of many ant species [39]. Such hybrid introgression in social hymenopterans has only been observed in the Africanized honey bee which resulted from the hybridization between two honey bee subspecies *Apis mellifera* × *A. m. scutellata* [40]. However, it is unknown if these findings in social hymenopterans are applicable to termites that are not haplodiploids.

### 3.3. Hybridization in Other Termites

The diplodiploid mating system in termites may change the potential hybridization outcome as observed in ants. However, only a few studies addressed gene flow among species in termites. Introgressive hybridization in termites was only investigated among populations of subspecies of the lower termites *Zootermopsis nevadensis* [41] and in *Reticulitermes lucifugus* [42]. Gene flow between different termite subspecies populations were confirmed in these two studies, showing that reticulate evolution occurs in termites and that determining maternal lineages using only mitochondrial markers reflects a partial aspects of termite species radiation. Conversely, no hybrid viability was obtained in laboratory experiments between two species of the fungus-growing termite *Pseudacanthotermes* [43], but hybrid viability was observed between two species of *Nasutitermes* for 60 d in the laboratory, although the success of these incipient colonies was not investigated further [44]. The genetic distance observed among hybridizing population in the field or in the laboratory with viable F_1_ was relatively small (<30 substitutions in the COII gene). In comparison, *C. gestroi* and *C. formosanus* populations in Florida have 78 substitutions for the COII gene [8] revealing a genetic distance between both species beyond the one previously observed in other hybridization events in termites.

## 4. Future Prospects on Hybridization in *Coptotermes*

**Figure 3:**
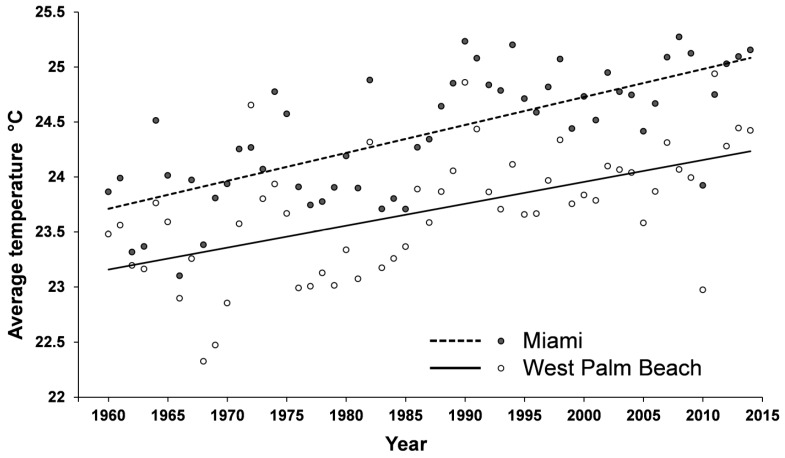
Changes in yearly average temperature in Miami and West Palm Beach over time. Source: NOAA.

The future evolutionary trajectory of the potential hybridization between the two *Coptotermes* species may depend on their intrinsic ecological requirement. As their overlapping distribution range has been increasing over the past decades, interspecies mating has become inevitable, as observed in other biological systems [45]. Chances for field establishment of hybrid colonies in the field may steadily increase in the years to come, and their detection may simply be a matter of time and adequate surveys at specific locations. A closer look at the distribution of *C. gestroi* in Taiwan, Hawaii and Florida suggests that the northern expansion by this tropical species is most likely a recent event. *Coptotermes gestroi* was reported from north of the Tropic of Cancer to central Taiwan only after the early 2000s, and currently is found in the northern tip of the island. Despite the earlier reports, *C. gestroi* infestation found in 1999 in Ewa Beach of Hawaii is probably the first establishment of this species on the island, and it has steadily expanded its distribution since then. Before 2000, *C. gestroi* in Florida was found only in Dade County and remained allopatric to *C. formosanus* until 2001 when a single alate was found in Broward County. Since then it has steadily expand northward to Palm Beach County. One factor affecting the northern expansion by *C. gestroi* may be the overall rise of temperature in these areas including south Florida in recent years (Figure 3).

Cao and Su [46] demonstrated that *C. gestroi* survived better at 20–35 °C than at <15 °C, while *C. formosanus* survived better at 15–30 °C than at >35 °C, which account for their geographic distribution. With the rise of temperature, it is expected that northern expansion by *C. gestroi* will continue, and if hybrid populations are found from our field collection, the potential hybrid zone will also expand northward. Moreover, our preliminary study showed that *C. formosanus* × *C. gestroi* hybrids possess temperature tolerance of both species, and survived well at 15–35 °C. If it turns out that hybrid alates are fertile and gene introgression occurs, there is a possibility that over evolutionary time, the hybrid zone in the new world alone may include the entire geographic range of both parent species, i.e., from Brazil to North Carolina.

Studies are underway to investigate temperature preference, colony growth rate, wood-consumption rate, insecticide susceptibility, fecundity, and other relevant characteristics of hybrid populations in comparison to the parent species. In Florida, both species are invasive and possess relatively low genetic diversity owing to their putative limited number of introduction [8]. With the restoration of heterozygocity in hybrid termites, and the ability of colonies to produce individuals with novel gene combinations, we expect some of the hybrid colonies to be highly variable. It is also expected that the selection process will eventually weed out the less vigorous populations, leaving the fittest to survive, and the survived hybrids may be more temperature extreme tolerant, grow faster, consume more wood and potentially cause major structural damage. If heterosis and F_1_ fertility is confirmed in hybrid *Coptotermes*, we also expect a rapid spread of hybrid populations beyond currently establish areas and gene introgression among the two species would be inevitable. We hope to answer these questions in the coming years, and confirm if hybrid termites have the potential for introgressive hybridization, with all its consequences, or if it is an evolutionary dead-end.

## 5. Conclusions

The status of *Coptotermes* hybrid populations is currently uncertain in sympatric locations. The one thing we know is that interspecies mating occurred in the field in Florida each year since at least 2013, and that such mating results in vigorous incipient colonies in the laboratory [8]. The fertility of F_1_ alates from such hybrid colonies remains to be determined, although obtaining swarming hybrid colonies in the laboratory may be challenging as it takes 5–10 years for an incipient colony to mature and produce winged reproductives, and confirmation of hybrid fertility by using these laboratory colonies may be time consuming. Once such F_1_ alates are produced in the laboratory, it will then be possible to test for their fertility by backcrossing them with their parental species and with other hybrid F_1_ individuals. However, even if winged reproductives of hybrids are sterile and a hybrid *Coptotermes* colony cannot initiate gene introgression among populations in the field, it can still live up to 15–20 years, may contain millions of workers, and can cause severe damage to nearby houses. We hypothesize that if the hybrid populations are fertile, then we should be able to collect hybrid alates from the field in the coming years, especially in places such as Southern Taiwan where these two species may have been sympatric for decades. Currently, we are planning to test this hypothesis with alates collected there. In addition, a large scale genetic population study on field collected *Coptotermes* in Florida and Taiwan is underway to monitor, in real-time, the potential establishment of hybrid colonies in the field.
